# Ginsenoside Rg1-Notoginsenoside R1-Protocatechuic Aldehyde Reduces Atherosclerosis and Attenuates Low-Shear Stress-Induced Vascular Endothelial Cell Dysfunction

**DOI:** 10.3389/fphar.2020.588259

**Published:** 2021-01-25

**Authors:** Lei Zhang, Yuan Li, Xin Ma, Jiali Liu, Xiaojie Wang, Lingxiao Zhang, Chao Li, Yunlun Li, Wenqing Yang

**Affiliations:** ^1^ First Faculty of Clinical Medicine, Shandong University of Traditional Chinese Medicine, Jinan, China; ^2^ Experimental Center, Shandong University of Traditional Chinese Medicine, Jinan, China; ^3^ Key Laboratory of Traditional Chinese Medicine Classic Theory, Ministry of Education, Shandong University of Traditional Chinese Medicine, Jinan, China; ^4^ Shandong Provincial Key Laboratory of Traditional Chinese Medicine for Basic Research, Shandong University of Traditional Chinese Medicine, Jinan, China; ^5^ Faculty of Traditional Chinese Medicine, Shandong University of Traditional Chinese Medicine, Jinan, China; ^6^ Cardiovascular Department, Affiliated Hospital of Shandong University of Traditional Chinese Medicine, Jinan, China

**Keywords:** atherosclerosis, endothelial dysfunction, piezo1, ginsenoside Rg1-notoginsenoside R1-protocatechuic aldehyde, shear stress

## Abstract

**Background:** The Fufang Danshen formula is a clinically important anti-atherosclerotic preparation in traditional Chinese medicine. However, its anti-atherosclerotic effect is not well recognized, and the mechanisms of its combined active ingredients, namely Ginsenoside Rg1-Notoginsenoside R1-Protocatechuic aldehyde (RRP), remain unclear. The purpose of this study was to investigate the anti-atherosclerotic effects and potential mechanism of RRP in ApoE^−/−^ mice and in low-shear stress-injured vascular endothelial cells.

**Methods: **ApoE^−/−^ mice were randomly divided into three groups: model group, rosuvastatin group, and RRP group, with C57BL/6J mice as the control group. Oil-red O, hematoxylin and eosin, Masson, and Movat staining were utilized for the observation of aortic plaque. Changes in the blood lipid indexes were observed with an automatic biochemistry analyzer. ET-1, eNOS, TXA_2_, and PGI_2_ levels were analyzed by enzyme-linked immunosorbent assay. *In vitro*, a fluid shear stress system was used to induce cell injury. Piezo1 expression in HUVECs was silenced using siRNA. Changes in morphology, proliferation, migration, and tube formation activity of cells were observed after RRP treatment. Quantitative Real-Time PCR and western blot analysis were employed to monitor mRNA and protein expression.

**Results: **RRP treatment reduced the atherosclerotic area and lipid levels and improved endothelial function in ApoE^−/−^ mice. RRP significantly repaired cell morphology, reduced excessive cell proliferation, and ameliorated migration and tube formation activity. In addition, RRP affected the FAK-PI3K/Akt signaling pathway. Importantly, Piezo1 silencing abolished the protective effects of RRP.

**Conclusion: **RRP has anti-atherosclerotic effects and antagonizes endothelial cell damage via modulating the FAK-PI3K/Akt signaling pathway. Piezo1 is a possible target of RRP in the treatment of atherosclerosis. Thus, RRP has promising therapeutic potential and broad application prospect for atherosclerosis.

## Introduction

In recent years, with the improvement of life standards and adjustment of diet structure, the incidence of coronary heart disease and ischemic stroke has increased, leading to higher risk of major adverse cardiovascular events (MACEs) (Sanchis-Gomar et al., 2016). MACEs are closely related to atherosclerotic plaque progression and rupture. Atherosclerosis is a chronic inflammatory disease characterized by dyslipidemia, foam cell formation, and lipid plaque accumulation in the artery wall (Li et al., 2016; Furuuchi et al., 2018). Vascular endothelial cells (VECs) are an important locus of critical regulatory nodes in the homeostatic network of the cardiovascular system (Szewczyk et al., 2015; Drozdz et al., 2018). Endothelial cell dysfunction within the arterial walls is an important contributor to the local and systemic manifestations of atherosclerotic cardiovascular disease (Gimbrone and Garcia-Cardena, 2013; Shimomura et al., 2018). Fluid mechanical forces generated by arterial blood flow could act directly on endothelial cells to alter their morphological and functional properties. Several studies suggest that distinct hemodynamic forces might constitute a local risk factor for endothelial cell dysfunction in atherogenesis (Gimbrone and Garcia-Cardena, 2016). Thus, shear stress sensors are a potential target to identify new anti-atherosclerotic drugs.

Phosphatidylinositol 3-kinase (PI3K) is a critical component of the signal transduction in the growth factor receptor superfamily. PI3K can be activated by a variety of cytokines as well as physical and chemical factors. PI3K phosphorylates and activates downstream kinases such as Akt, protein kinase C, and phosphoinositide-dependent kinase 1. Among these, Akt, which activates endothelial nitric oxide synthase (eNOS) to produce nitric oxide (NO), is the most important (Rosenberg et al., 2018). PI3K/Akt signaling pathway plays a key role in various cellular processes, including cell survival, growth, and proliferation (Chen et al., 2015). In addition, the PI3K/Akt complex can be used as a biomarker with predictive and prognostic values (Sadeghi and Gerber, 2012; Hiob et al., 2016). Piezo1 is a novel mechanically activated ion channel that has the ability to sense mechanical signals and regulate cell volume homeostasis (Faucherre et al., 2014). Piezo1 is widely expressed in VECs and plays a crucial role in the regulation of cardiovascular development and physiological function. Piezo1 can be activated by shear forces caused by blood flow (Ranade et al., 2014). Laminar shear can activate Piezo1 to regulate the release of NO from endothelium, thus affecting the local vascular tension and playing an anti-atherosclerotic role (Wang et al., 2016). However, if the VECs are affected by eddy currents, activated Piezo1 will promote atherosclerosis development through the NF-κB pathway (Hahn and Schwartz, 2009; Albarran-Juarez et al., 2018).

**FIGURE 1:**
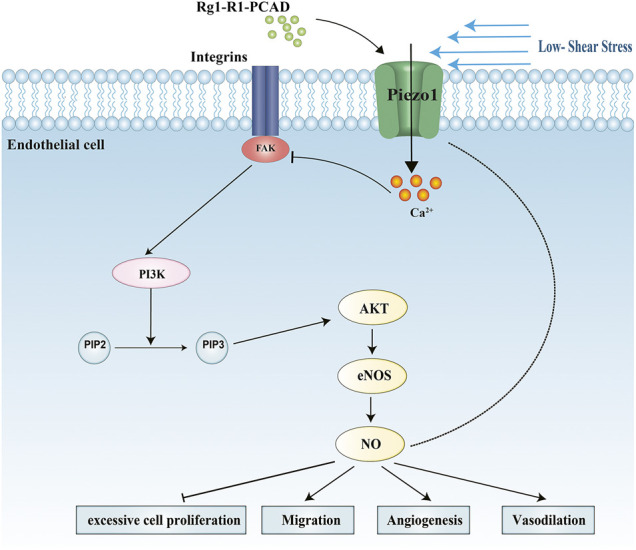
Schematic model of the protective effects of RRP on endothelial cells against low-fluid shear stress-induced injury.

Fufang Danshen formula is a clinically important anti-atherosclerotic drug. Ginsenoside Rg1 (Rg1), Notoginsenoside R1 (R1), and Protocatechuic aldehyde (PCAD), collectively referred to as RRP, are its main active components with anti-atherosclerotic action. We have previously used the active components to antagonize endothelial cell damage and showed that its protective functions may relate to the reduction of the inflammatory reaction (Qi et al., 2012; Yang and Yao, 2012). However, the mechanism by which RRP protects endothelial cells is not clear. Based on the previous research, here we investigated the effect of RRP on atherosclerosis in ApoE^−/−^ mice. To further elucidate the mechanisms, we hypothesized that the protective effect of RRP on endothelial cells may be related to Piezo1. We aimed to prove that RRP could antagonize endothelial cell damage by acting on Piezo1. Therefore, we examined the protective effects of RRP on endothelial cells against low-fluid shear stress-induced injury. Our results suggest that RRP has anti-atherosclerotic effect and antagonizes endothelial cell damage via influencing the FAK-PI3K/Akt signaling pathways. Our data also implicate that Piezo1 is a possible target of RRP in the treatment of atherosclerosis (Figure 1). These results indicate that RRP has promising therapeutic potential and broad application prospect for atherosclerosis.

## Materials and Methods

### Preparation of Rg1, R1, and PCAD

Rg1 (Dalian Meilun Biotechnology Co., LTD. China), R1 (Shanghai standard biotechnology, China), and PCAD (Dalian Meilun Biotechnology Co., LTD. China) were dissolved in trace dimethyl sulfoxide and then diluted to stored concentration with DMEM·F12. The solution was stored at −20°C in the dark.

### Animal Experiments

Male ApoE^−/−^mice (23.12 ± 1.18 g) and male C57BL/6 mice (22 ± 2.2 g), both aged 8 weeks, were purchased from Beijing Weitonglihua Experimental Animal Technology co., Ltd. (Beijing, China). The animal certificate number was SCXK (Jing) 2014-0004. The animal experiments were performed in accordance with the Guide for the Care and Use of Laboratory Animals (published by the US National Institutes of Health) and approved by the Institutional Animal Care and Research Advisory Committee of the Shandong University of Traditional Chinese Medicine. All mice were maintained under SPF laboratory conditions with water and food ad libitum, at a temperature of 22 ± 2°C on a 12 h light/dark cycle during the experimental period. After 1 week of adaptation, all mice were fed with a high-fat diet including 0.15% cholesterol and 21% pork fat. After 12 weeks of continuous high-fat diet, ApoE^−/−^ mice were randomly divided into three groups (*n* = 10): model group (receiving vehicle, NaCl, i.p.); rosuvastatin group (positive-control group, 10 mg kg^−1^, i.g.), and RRP group ([10 mg Rg1 + 10 mg R1 + 14 mg PCAD]·kg^−1^, i.p.). Ten C57BL/6J mice were used as control group (vehicle; NaCl, i.p.). All animals received treatment once daily continuously for 8 weeks.

### Specimen Collection and Processing

After 8 weeks of intervention, mice were weighed, fasted overnight, and anesthetized using isoflurane. Fresh blood samples were drawn from the thoracic aorta. Serum was centrifuged at 1,301 × *g* for 10 min at 4°C. After the blood collection, the thoracic and abdominal cavity was quickly opened to expose the heart and perfuse the cardiovascular system. The aortic root and part of the myocardial tissue were taken and fixed in 4% paraformaldehyde (PFA). Aorta samples were removed and stored at −80°C or soaked in 4% PFA.

### Pathological Morphology Analysis

The en face aorta was stained with Oil-red O and to assess overall burden and distribution of atherosclerosis. Briefly, whole aortas were opened longitudinally, and fixed with 4% PFA solution for 24 h, followed by a 3 min rinse with PBS. Then, the whole aortas were stained with 0.5% Oil-red O working solution for 60 min at 37°C in a biochemical incubator. Subsequently, whole aortas were immersed into 70% ethanol for destaining and then rinsed with PBS. The images were captured by a stereomicroscope. The extent of aortic atherosclerosis was evaluated as the ratio of lesion area to aortic area.

Subsequently, the aortic sinus was fixed with 4% PFA solution for 24 h for later paraffinization. The aortic sinus was sliced into 5 µm serial paraffin sections. Hematoxylin and eosin (H&E), Masson, and Movat staining (at room temperature, RT) were performed on the sections to determine aortic lipid plaque areas, collagen fiber content, and elastin plaque area respectively. Images were collected under a microscope (ZEISS, Germany) and measured by the Image-pro plus 6.0 (Media Cybernetics, United States).

### Determination of Serum Lipid Concentration

Serum concentrations of total cholesterol (TC), triglyceride (TG), high-density lipoprotein cholesterol (HDL-C), and low-density lipoprotein cholesterol (LDL-C) were detected by a ZY-310 Automatic Biochemistry Analyzer (Shanghai Kehua Bio-engineering, Shanghai, China) according to the manufacturer's instructions. The atherosclerosis index (AI) was derived as AI = non-HDL-C/HDL-C.

### Determination of Serum Biochemical Parameters

Serum concentrations of eNOS, endothelin-1 (ET-1), Prostaglandin I2 (PGI_2_), and Thromboxane A2 (TXA_2_) were determined by enzyme-linked immunosorbent assay (ELISA) kits (Elabscience Biotechnology, Wuhan, China). All operations followed the manufacturer’s protocols.

### Cell Culture

The human umbilical vein endothelial cell (HUVEC) line (8,000) was purchased from ScienCell Research Laboratories (Carlsbad, CA). HUVECs were cultured in endothelial cell medium (ECM) supplemented with 5% FBS, 1% ECGS, and 1% P/S Solution (ScienCell, California, United States) at 37°C and 5% CO_2_. Except for the static culture group (cultured only with ECM), cells in other groups were treated as described below.

### Shear Stress Experiments

An Ibidi pump system (Ibidi, Munich, Germany) was used to provide low-fluid shear stress (L-FSS). HUVECs were seeded onto a µ-Slide I 0.4 Luer (Special channel slides of Ibidi) and incubated for 24 h to form a monolayer. Laminar shear stress was created by attaching channel slides to a peristaltic pump. The experiment was conducted under the following conditions: 6-mbar pressure, 2.5 ml min^−1^ flow rate, and a shear stress of 4 dyn/cm^2^ HUVECs in the control group were exposed to shear stress for 24 h. The drug intervention group was cultured with RRP [(Rg1 100 μg + R1 100 μg + PCAD 30 μg) ·mL^−1^] for 1 h before being exposed to shear stress.

### siRNA Transfection

HUVECs were transfected with 50 nM Piezo1 siRNA. According to the manufacturer’s specification, siRNA and the transfection reagent were added to the siRNA transfection medium and then incubated ∼10–30 min at RT. The cells were incubated with the siRNA transfection reagent mixture for 6 h. The transfection mixture was then carefully removed, and complete medium was added for 48 h for subsequent experiments. The expression of Piezo1 was assessed to evaluate the efficiency of transfection. Cells transfected with Piezo1 siRNA were used as control. The other groups were exposed to shear stress with or without RRP [(Rg1 100 μg + R1 100 μg + PCAD 30 μg) ·mL^−1^] for 24 h.

### Immunofluorescence

F-actin expression in HUVECs was examined by immunofluorescence to observe cell morphology. Cells grown on channel slides were fixed with 4% PFA for 10 min at RT, followed by three washes with PBS. Cells were then permeated with 0.1% Triton X-100 for 5 min and blocked with 1% bovine serum albumin (BSA) for 30 min. F-actin antibody (KeyGEN BioTECH, Jiangsu, China, 1:40 dilution) was added and incubated in the dark for 20 min at RT. Following washes with PBS, the nuclei were stained with DAPI (Abcam, USA) in the dark for 5 min at RT. Cells were observed and images collected under a microscope (ZEISS, Germany).

### Proliferation Assay

The CCK-8 assay was used to detect HUVEC proliferation ability. According to the manufacturer’s instructions, 100 μl culture medium and 100 μl cell suspension (8 × 10^4^ cells/ml) were added to 96-well plates. After different treatments, the culture medium was aspirated, and cells were then incubated with CCK-8 reagent for 1 h at 37°C. Absorbance was measured at 450 nm by a microplate reader (BioTek, United States).

### Migration Assay

A Transwell chamber (Coning, United States) was used to determine HUVEC migration rate. HUVECs were diluted to 1 × 10^5^ cells/ml with serum-free medium and 200 μl cell suspension was added to the upper compartment. ECM medium (0.5 ml) containing 5% FBS was added to the lower chamber. The upper chamber was placed in the lower chamber and incubated for 6 h at 37°C. Using a dry cotton swab to wipe the remaining cells on the upper chamber, the cells on the underside of the upper chamber membrane were fixed with 4% PFA for 30 min. Then, the cells were stained with H&E for 30 and 10 min, respectively. Images were collected under a microscope (ZEISS, Germany) and the number of HUVECs that had migrated was counted using the Image-pro plus 6.0 (Media Cybernetics, United States).

### Tube Formation Assay

HUVEC tube formation was performed using an *In Vitro* Angiogenesis Tube Formation Assay Kit (Trevigen, United States). Each well of a 96-well plate was coated with 50 μl of BME at 4°C and polymerized for 1 h at 37°C. HUVECs were diluted to 1 × 10^4^/ml with serum-free medium and 100 μl of cell suspension was added to the top of the gel and incubated for 4 h. Then, the culture medium was aspirated and cells were washed with PBS before adding 100 μl of Calcein AM (2 μM) into each well. The formation of capillary-like tubes was observed after 30 min. Images were collected under a microscope (ZEISS, Germany), and the number of junctions and segments and the length of the network were calculated using the Image-pro plus 6.0 (Media Cybernetics, United States).

### Western Blot Analysis

Protein extracts of cells were obtained using cell lysis buffer (Beyotime Institute of Biotechnology, Shanghai, China) with protease inhibitors (1:100); the supernatant was collected after centrifugation at 15,294 × *g* for 15 min at 4°C. Protein concentration was assayed using a BCA Protein Assay Kit (Dalian Meilun Biotechnology Co., Ltd, Dalian, China). Thirty micrograms of proteins per sample were separated by 10% sodium dodecyl sulfate-polyacrylamide gel electrophoresis (SDS-PAGE) and transferred onto polyvinylidene fluoride (PVDF) membranes (Millipore corporation, United States). The membranes were blocked with TBST containing 5% skim milk for 1 h, and then incubated overnight at 4°C with specific primary antibodies against AKT (Cell Signaling Technology), PI3K (Cell Signaling Technology), FAK (Cell Signaling Technology), eNOS (Abcam), and β-actin (Proteintech). The membranes were rinsed with TBST for three times and then exposed to suitable secondary HRP-conjugated antibodies at RT for 1.5 h. ECL (Millipore corporation, United States) was used for signal detection. Blot data were analyzed with Image-Pro Plus 6.0 (Media Cybernetics, United States).

### Quantitative Real-Time PCR

Total RNA was extracted from HUVECs with RNAprep Pure Micro Kit (Tiangen Biotech [Beijing] Co., Ltd, Beijing, China) according to the manufacturer’s protocol. Total RNA was quantified with NanoDrop One (Thermo Fisher Scientific, United States). Total RNA (1 μg) was reverse-transcribed into cDNA using 5X All-In-One RT MasterMix (Tiangen Biotech [Beijing] Co., Ltd, Beijing, China). qRT-PCR was performed with TB Green™ Premix Ex Taq™ II kit (Takara, Japan). The expression of specific genes was measured and analyzed by QuantStudio 5 (Thermo Fisher Scientific, United States). The sequences of primers used, from 5' to 3' extremity, were as follows:

AKT: CTG​CAC​AAA​CGA​GGG​GAG​TA (F), GCG​CCA​CAG​AGA​AGT​TGT​TG (R); PI3K: CCC​AGG​TGG​AAT​GAA​TGG​CT (F), GCC​AAT​GGA​CAG​TGT​TCC​TCT (R);

FAK: GCT​CCC​TTG​CAT​CTT​CCA​GT (F), AAT​ACT​GGC​CCA​GGT​GGT​TG (R);

eNOS: GCC​GGA​ACA​GCA​CAA​GAG​TTA​T (F), AGC​CCG​AAC​ACA​CAG​AAC​C (R);

β-actin: CTC​ACC​ATG​GAT​GAT​GAT​ATC​GC (F), AGG​AAT​CCT​TCT​GAC​CC-ATG​C (R).

### Statistical Analysis

Data were analyzed using SPSS software (version 22.0; IBM, Armonk, NY, United States) and are expressed as the mean ± SEM. Multiple-group comparisons were analyzed using one-way analysis of variance followed by Tukey’s post-hoc multiple range test. *p* < 0.05 was considered to indicate a statistically significant difference.

## Results

### RRP Administration Significantly Alleviates the Development of Atherosclerosis

**FIGURE 2:**
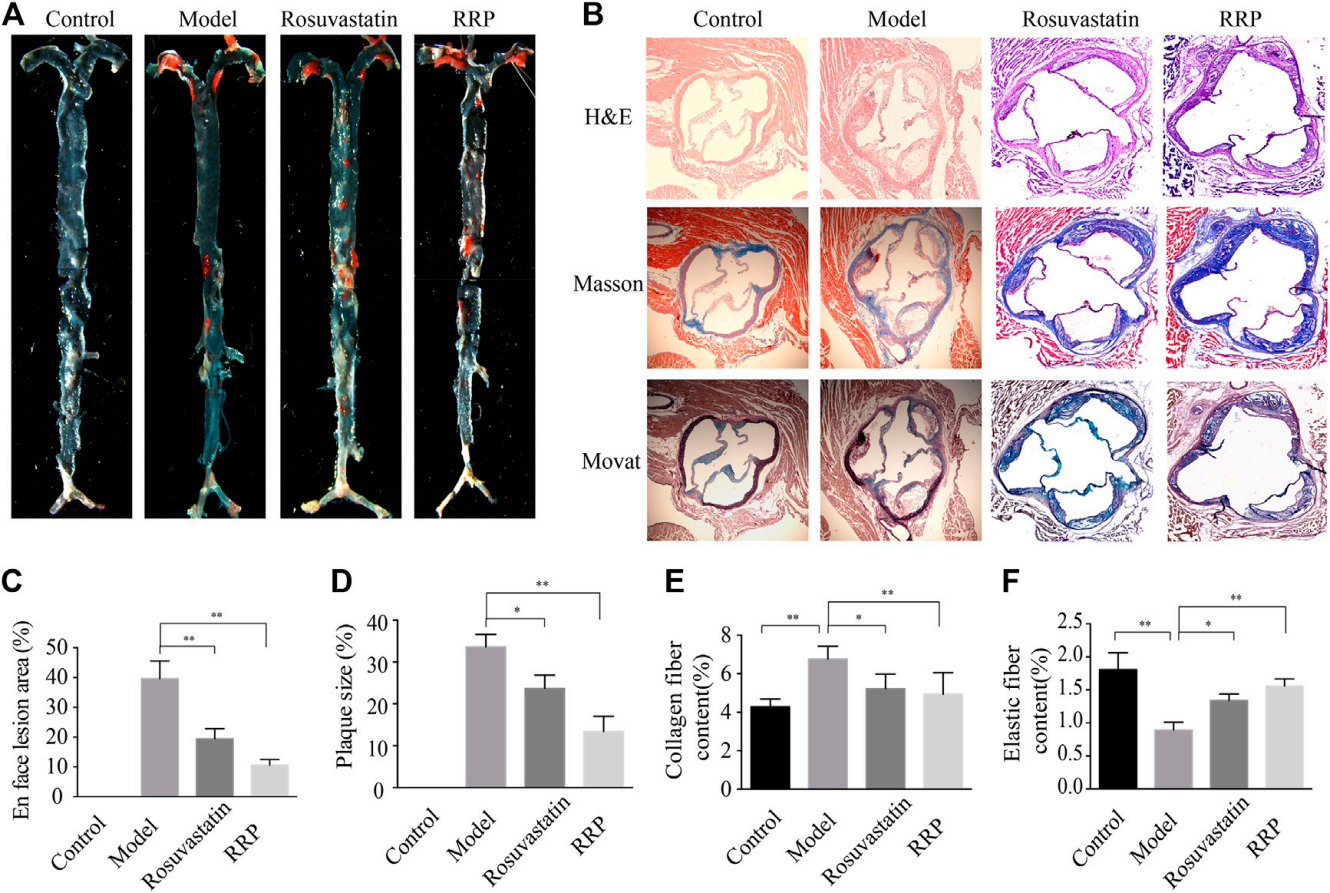
RRP affects the development of atherosclerosis. **(A)** Representative Oil-red O staining of the entire aorta. **(B)** Representative images of H&E, Masson, and Movat staining of the aortic root (magnification, ×50). **(C)** Quantification of the en face lesion area of the entire aorta. **(D)** Percentage of aortic plaque area. **(E)** Percentage of collagen fiber content. **(F)** Percentage of elastin plaque area (elastin per plaque). The data are expressed as the mean ± SD, *n* = 6 for each group. ∗*p* < 0.05 vs. the model group.

**FIGURE 3:**
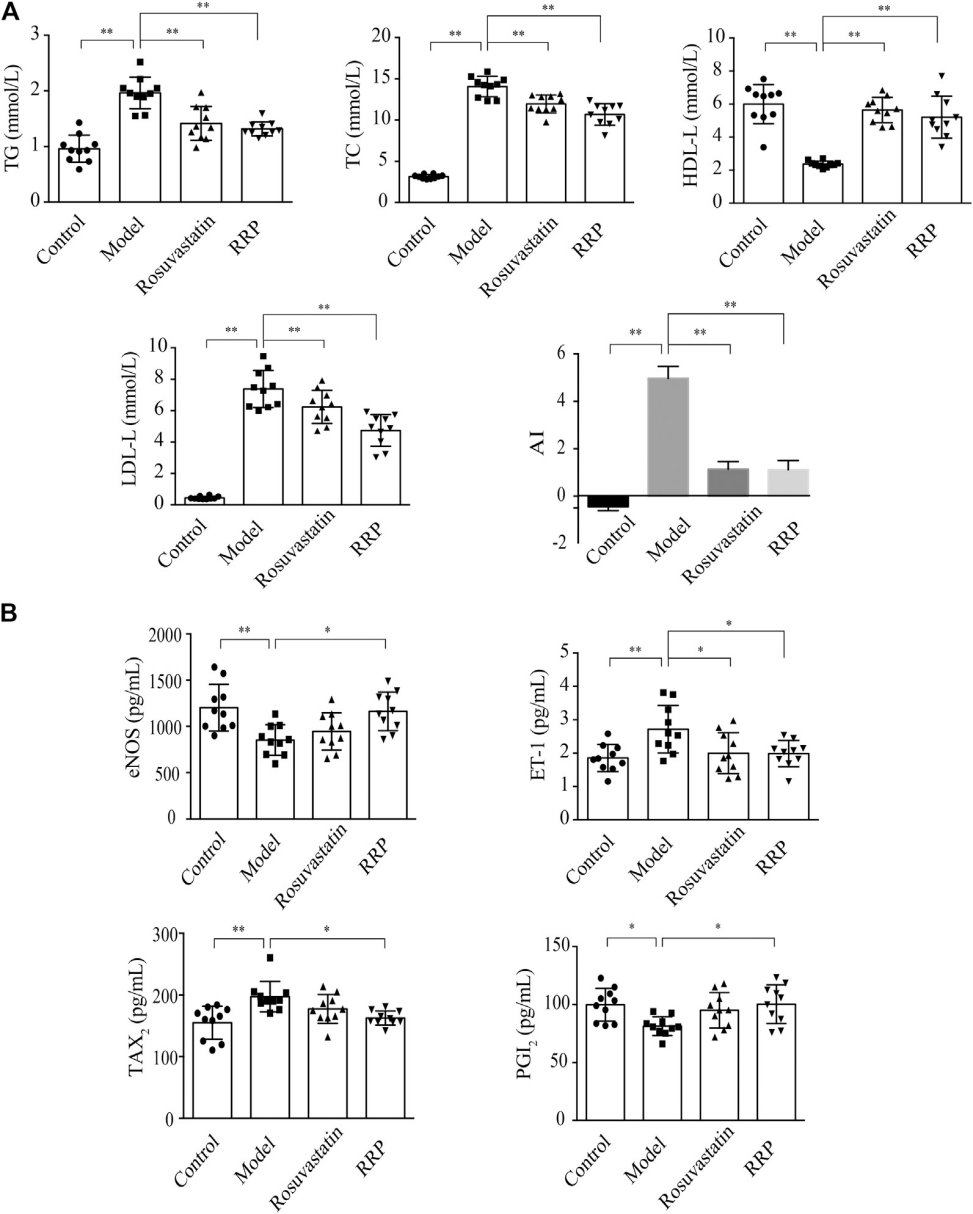
RRP influences lipids and secretion of vessel endothelial factors in serum. **(A)** Serum levels of lipids (TG, TC, LDL-C, and HDL-C) in mice. **(B)** Levels of eNOS, ET-1, PGI_2_, and TXA_2_ in mice. The data are expressed as the mean ± SD, *n* = 10 for each group. ∗*p* < 0.05, ∗∗*p* < 0.001 vs. the model group.

We examined whether RRP can affect overall burden and distribution of atherosclerosis in order to assess the inhibitory effect of RRP on the progression of atherosclerotic lesions. The oil-red O-positive area in the model group, particularly the area of the aortic arch, was significantly higher than in the control group (Figures 2A,C). However, RRP treatment notably reduced the atherosclerotic plaque size in the whole aorta. The severity of atherosclerosis was evaluated with H&E, Masson, and Movat staining of paraffin sections of the aortic sinus. As shown in Figures 2B,D–F, the aortic roots of model group mice exhibited significant formation of atherosclerosis plaque, and a mass of foam cells and cholesterol crystals in the plaque. The aorta intima displayed serious lesions and decrease in collagen and elastic fibers to various degrees. Encouragingly, RRP treatment repressed the pathological changes in the aorta, inducing similar improvements to rosuvastatin treatment. In addition, we measured the serum lipid levels in mice. In ApoE^−/−^ mice, TC, TG, LDL-C, and AI levels were dramatically higher than in control mice, while HDL-C was reduced. However, RRP could improve the lipid index to different degrees (Figure 3A). These results show that RRP has beneficial functions on controlling dyslipidemia.

### The Regulatory Effects of RRP on Secretion Function of Vessel Endothelium in Serum.

Impaired endothelial function is a key point in initiation and development of atherosclerosis (Cancel et al., 2016). Therefore, we next determined the effect of RRP in improving vascular endothelial function. ET-1 and NO are important regulators of cardiovascular system homeostasis. Under pathological conditions, the production of NO decreases following changes in eNOS activity, leading to the occurrence and development of cardiovascular diseases. In the model group, ET-1 was significantly up-regulated and eNOS significantly down-regulated (*p* < 0.01). However, the concentration of ET-1 was reduced by both RRP and rosuvastatin administration (*p <* 0.05) (Figure 3B). RRP also increased eNOS content (*p <* 0.05), but no change was observed with rosuvastatin. PGI_2_ and TXA_2_ are vasoactive substances released by vascular endothelial cells. The balance between PGI_2_ and TXA_2_ is an important influence on vascular wall tension. The imbalance between PGI_2_ and TXA_2_ could also be restored by RRP but not rosuvastatin (Figure 3B).

### RRP Ameliorates Low-Shear Stress-Induced Cell Function Damage

**FIGURE 4:**
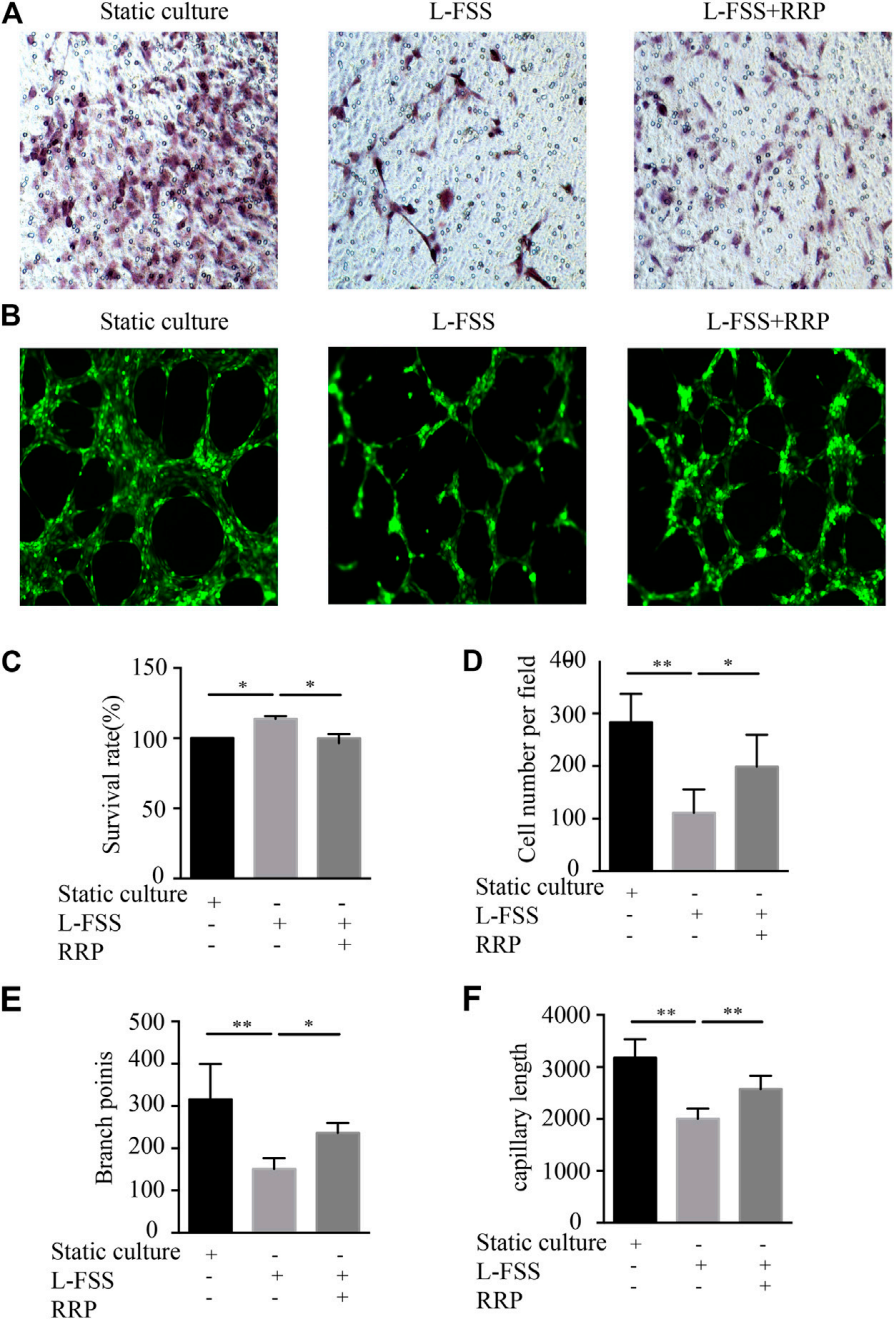
RRP affects HUVEC proliferation, migration, and tubule formation. **(A,D)** Representative images of the migration of HUVECs and the calculated number of cells that migrated, scale bars = 50 μm, *p < 0.05, **p < 0.001 vs. L-FSS group. **(B)** Representative images of the formation of capillary-like tubules in HUVECs, scale bars = 200 μm. **(C)** Proliferation activity of HUVECs, *p < 0.05 vs. L-FSS group. **(E,F)** Capillary morphogenesis was quantified by calculating the branch points **(E)** and capillary length **(F)**, *p < 0.05, **p < 0.001 vs. L-FSS group.

We used a fluid shear system to induce VEC injury *in vitro*, in order to explore whether RRP can antagonize L-FSS-induced cell function damage. We first observed the effect on cell proliferation (Figure 4C). L-FSS induced excessive cell proliferation, which was inhibited by treatment with RRP. Next, we evaluated the effects of RRP on endothelial cell migration (Figures 4A,D). We observed that migration of HUVECs decreased under L-FSS conditions (*p* < 0.01) but increased after RRP treatment (*p* < 0.05). Finally, to clarify the effect on angiogenic potential, we examined the formation of capillary-like tubules (Figures 4B,E,F). Compared to the L-FSS group, the RRP group showed significantly higher tube formation activity. These results indicate that cellular functions damaged by L-FSS could be improved by RRP.

### Piezo1 Depletion Offsets the Protective Effect of RRP on HUVECs

**FIGURE 5:**
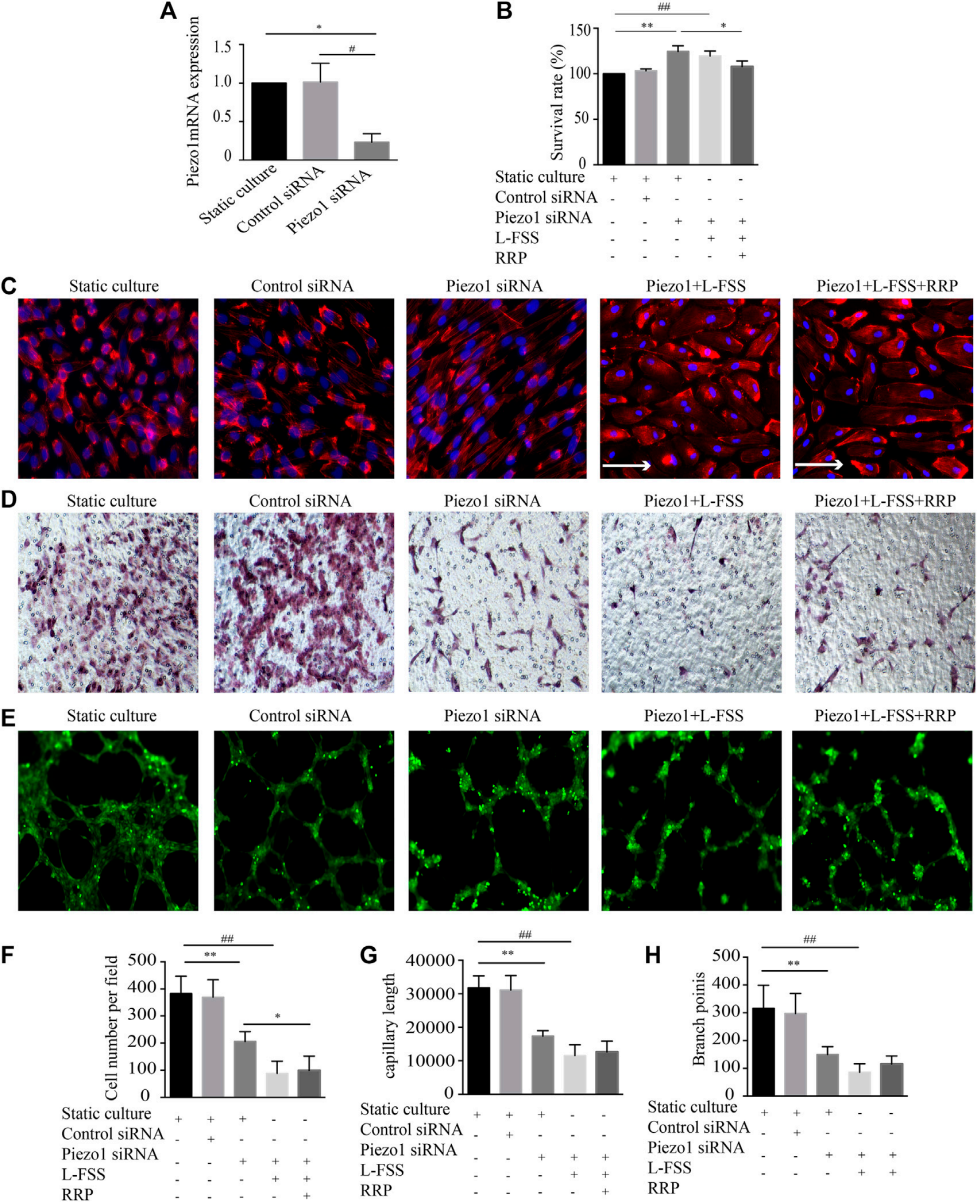
Inhibition of Piezo1 using siRNA abrogates the protective effects of RRP on HUVEC injury. **(A)** The mRNA expression of Piezo1 detected by qRT-PCR in indicated groups. **(B)** Proliferation activity of HUVECs, *p < 0.05 vs. Piezo1 siRNA group. **(C)** Cellular alignment was analyzed by staining cells with phalloidin (red) and DAPI (blue), scale bars = 50 μm, arrows indicate the fluid direction. **(D,F)** Representative images of the migration of HUVECs and the calculated cell migration number, scale bars = 50 μm, *p < 0.05, **p < 0.001 vs. Piezo1 siRNA group, ^##^ *p* < 0.001 vs. Piezo1 siRNA + L-FSS group. **(E)** Representative images of the formation of capillary-like tubules in HUVECs, scale bars = 200 μm. **(G,H)** Capillary morphogenesis was quantified by calculating the branch points **(G)** and capillary length **(H)**, **p < 0.001 vs. Piezo1 siRNA group, ^#^ *p* < 0.05, ^##^ *p* < 0.001 vs. Piezo1 siRNA + L-FSS group.

Piezo1 can sense the change in shear force on VECs. To explore whether the protective effect of RRP was associated with Piezo1, we silenced expression of this channel in HUVECs by siRNA. Firstly, in Piezo1-silenced HUVECs, the morphology changed from cobblestone-like to spindle-shaped, and the cell arrangement changed from random to orderly (Figure 5A). Instead, Piezo1 siRNA + L-FSS cells changed morphology from cobblestone-like to irregular-shaped and showed aggregation of F-actin filaments, suggesting the occurrence of cytoskeletal rearrangement and cell damage (Figure 5C). Secondly, knockdown of Piezo1 in HUVECs stimulated cell proliferation (Figure 5B) and decreased cell migration (Figures 5D,F) and tubular structure (Figures 5E,G,H). We also detected increased cell damage in Piezo1 siRNA + L-FSS group. Concomitantly, we found that knockdown of Piezo1 remarkably abolished the protective effect of RRP. These results suggest that the protective effect of RRP against cellular function damage may be related to Piezo1.

### RRP Protects HUVECs by Activating the FAK-PI3K/Akt Signaling Pathway

**FIGURE 6:**
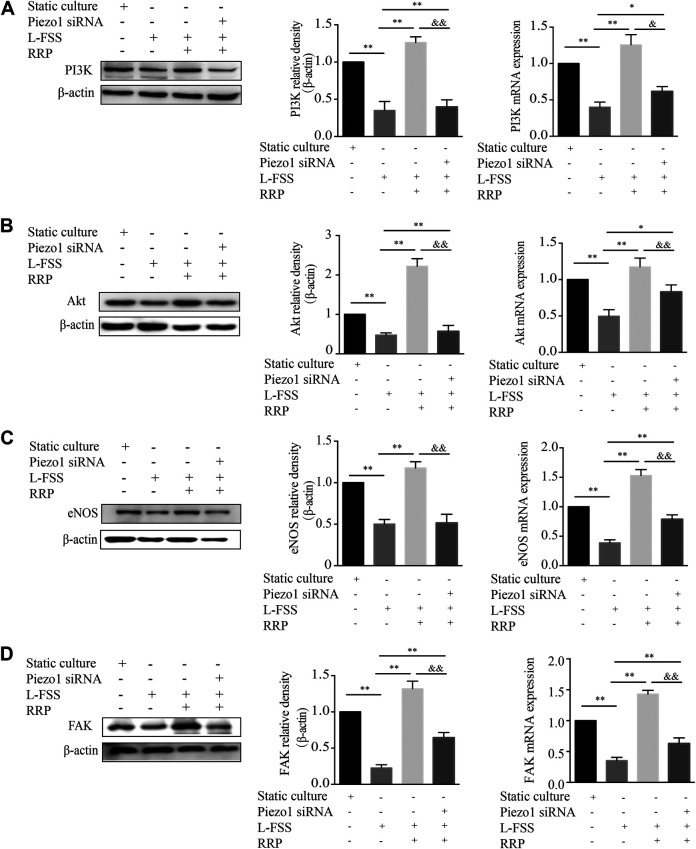
RRP intervenes in the FAK-PI3K/Akt pathway. The mRNA and protein levels of PI3K **(A)**, Akt **(B)**, eNOS **(C)**, and FAK **(D)** were detected by qPCR and western blot, respectively. *p < 0.05, **p < 0.001 vs. L-FSS group, ^&^ *p* < 0.05, ^&&^ *p* < 0.001 vs. L-FSS + RRP group.

The PI3K/Akt signaling pathway plays essential roles in the proliferation, migration and apoptosis of VECs (Shi and Feng, 2011; Polivka and Jnaku, 2014). It also plays an important role in improving endothelial function and reducing atherosclerosis (García-Prieto et al. 2015; Wang et al., 2015). Next, we detected the expression levels of PI3K and Akt in HUVECs. As shown in Figures 6A,B, the expression of PI3K and Akt was significantly decreased by L-FSS pretreatment. However, downregulation of PI3K and Akt was reversed in RRP-treated HUVECs. We also found that the activity of eNOS increased along with the higher expression of PI3K/Akt in L-FSS + RRP group (Figure 6C). PI3K has also been shown to bind FAK, leading to activation of PI3K and its downstream effectors (Seguin et al., 2015). Next, we investigated the effects of RRP on low-shear stress-induced expression of FAK. We found that FAK levels were significantly higher in RRP-treated compared to L-FSS group (Figure 6D). Together, these results suggest that RRP might protect HUVECs against L-FSS-induced injury by activating the FAK-PI3K/Akt pathway. Moreover, we silenced Piezo1 to assess whether the activation of FAK-PI3K/Akt pathway by RRP is related to this sensor. As shown in Figure 6, the activating effect of RRP was abolished to varying degrees in Piezo1-silenced cells. We can infer that Piezo1 is thus involved in RRP-mediated activation of FAK-PI3K/Akt signaling.

## Discussion

Atherosclerosis is a vascular chronic inflammatory disease resulting from lipid deposition in the vascular wall, with rupture-prone plaque and changes in structure and function of the vascular wall (Zamaklar et al., 2009; Taleb et al., 2014). Drugs that treat atherosclerosis usually act nonspecifically. The majority of patients receive statins, antiplatelet therapy, beta-blockers, or angiotensin-converting enzyme inhibitors/angiotensin receptor blockers (Vedin et al., 2013). However, these drugs have some side effects such as rhabdomyolysis and conduction block. Moreover, atherosclerosis is a complex disease with unknown etiology. Single-target therapy is therefore unlikely to be effective against atherosclerosis. Traditional Chinese medicine (TCM), with its characteristic syndrome differentiation, has unique advantages in the treatment or prevention of atherosclerosis. Several classic prescriptions or active ingredients of single herbs can effectively prevent atherosclerosis, reduce the incidence of cardiovascular events, and treat atherosclerosis, with a wide range of applications. The Fufang Danshen formula is a clinically important anti-atherosclerotic drug with the characteristics of multi-component, multi-target, and multi-channel. Numerous studies based on different target molecules have been conducted to dissect the exact mechanism of action of Fufang Danshen formula (Zhou et al., 2005). Due to the complex composition of Fufang Danshen formula, preliminary work of our group has determined Rg1, R1, and PCAD as the best combination of active ingredients in order to clarify the underlying anti-atherosclerotic mechanism. The current study demonstrates the anti-atherosclerotic effects of RRP in ApoE^−/−^mice. RRP significantly reduced atherosclerotic area and lipid level in ApoE^−/−^ mice. Our data also showed that the expression of ET-1 and TXA_2_ was decreased while that of eNOS and PGI_2_ was increased in response to RRP treatment. These results suggest that the endothelial damage is repaired in RRP-treated ApoE^−/−^ mice.

VECs appear to sense shear stress of the vascular cavity surface and transduce mechanical signals, which can regulate various signaling pathways and physiological functions (Baeyens et al., 2016). VEC dysfunctions have been found to play vital roles in the initiation of vascular disorders and atherosclerosis (Mundi et al., 2017) and lead to the earliest detectable changes in the life history of an atherosclerotic lesion (Stary, 2000; Virmani et al., 2000). Fluid shear stress is an important regulator of VEC functions (McCormick et al., 2012). Low-shear stress is an important risk factor for VEC injury (Man et al., 2018; Baeriswyl et al., 2019). In regions where the blood flow is multidirectional (low-fluid shear stress or disturbed flow), VECs are activated and atherosclerotic plaque is detected (Le and Abe, 2018). In the present study, we demonstrated that RRP ameliorated L-FSS-induced HUVEC dysfunctions. RRP also significantly repaired cell morphology, reduced cell excessive proliferation, and improved migration and tube formation activity.

PI3K/Akt signaling, like different vascular growth factors, regulates a variety of cellular processes, such as cell growth, proliferation, migration, metabolism, and angiogenesis (Cantley, 2002; Whiteman et al., 2002). Moreover, the PI3K/Akt pathway can mediate shear stress-induced signaling (Li et al., 2005). Interfering with the PI3K/Akt signaling pathway is an effective strategy to inhibit endothelial dysfunction. In this study, we confirmed that PI3K and Akt expression was increased after RRP treatment. PI3K/Akt are key enzymes controlling eNOS phosphorylation, and blocking PI3K/Akt can partially inhibit eNOS activity (Fulton et al., 2002; Fleming and Busse, 2003; Zhang et al., 2018). Akt can activate eNOS, which can cause the production of NO to promote VEC migration and angiogenesis and enhance vasodilation (Fu et al., 2013; Ahsan et al., 2015). In our study, the use of RRP regulated the expression of Akt and thus increased eNOS in low-shear stress-treated HUVECs. Moreover, FAK is a non-receptor cytoplasmic protein tyrosine kinase and is involved in many signal transduction pathways. FAK is a key regulator of cell proliferation, migration, and adhesion (Benelli et al., 2010). The activation of FAK is required for focal adhesion turnover and actin cytoskeletal dynamic reorganization in cell migration (Cheng et al., 2017). In addition, FAK has been shown to activate the PI3K/Akt signaling pathway (Horowitz et al., 2007; Garrison et al., 2013). Our results showed that RRP increased the level of FAK, suggesting that RRP promoted FAK and thereby enhanced expression of PI3K and Akt. Therefore, we deduced that RRP could play a protective role for VECs by intervening in the FAK-PI3K/Akt signaling pathway.

Shear stress, which is a major physiological stimulus, can promote VECs to release vasoactive factors and regulate vascular tone (Dehghan et al., 2016). VECs responsiveness to shear stress is essential for vasoregulation and plays a role in atherogenesis (Choi and Barakat, 2005). Piezo1, an essential component of mechanically activated channels, is a sensor of blood flow through shear stress-evoked ionic current and calcium influx in endothelial cells (Li et al., 2014). Piezo1 is an important target for regulating VEC function and maintaining cardiovascular homeostasis. Loss of Piezo1 in endothelial cells leads to decrease of the cell's response to shear stress, disorder of alignment in the direction of flow, and damaged cell function (Li et al., 2014; Ranade et al., 2014). Research has suggested that laminar shear stress can activate Piezo1 and modulate NO release from endothelium to maintain vascular tension of local blood vessels (Wang et al., 2016). Here, we found that Piezo1 siRNA could abolish the protective effects of RRP against cellular function damage, suggesting that these functions of RRP may be related to Piezo1.

Our study also has some limitations. There is a growing interest in therapy using Chinese medicine directed towards several targets/pathways. This study only focused on the effects of RRP on the FAK-PI3K/Akt signaling pathway and its influence on VEC function. However, its role in other signaling pathways related to the pathogenesis of atherosclerosis and other vascular cell types has not been fully explored. Moreover, although the efficacy of RRP is significantly affected by Piezo1 depletion, the specific underlying mechanism is still not clear.

## Conclusion

In summary, our study provided evidence that RRP exerts excellent effects on the complicated condition of atherosclerosis. RRP has a positive role in improving cellular function induced by L-FSS injury, which is achieved by modulating FAK- PI3K/Akt signaling, reducing excessive cell proliferation, and ameliorating migration and tube formation activity, thereby effectively inhibiting vascular injury. Finally, our study reveals Piezo1 as a possible target of RRP in the treatment of atherosclerosis. Our findings provide new insights for future therapeutic options for atherosclerosis.

## Data Availability Statement

The raw data supporting the conclusions of this article will be made available by the authors, without undue reservation, to any qualified researcher.

## Ethics Statement

The animal study was reviewed and approved by Animal Ethics Committee of Shandong University of Traditional Chinese Medicine.

## Author Contributions

LeZ and YuaL participated in all experimental work and drafted the paper. XM and XW conducted the *in vivo* experiments. LiZ conducted the *in vitro* experiments. LeZ, YuaL, and WY performed data analysis. CL arranged the figures. WY and YunL (8th author) designed the experimental protocols.

## Funding

This study was supported by grants from the NSFC Youth Science Foundation Project (81804006), National Natural Science Foundation of China (81974566) and Supported by China Postdoctoral Science Foundation (2020M672125). This study was also supported by the Program of Scientific research projects in Universities of Shandong Province (J18KA260).

## Conflict of Interest

The authors declare that the research was conducted in the absence of any commercial or financial relationships that could be construed as a potential conflict of interest.

## Abbreviations

MACEs, major adverse cardiovascular events; VECs, vascular endothelial cells; PI3K, Phosphatidylinositol 3-kinase; eNOS, nitric oxide synthase; NO, nitric oxide; Rg1, Ginsenoside Rg1; R1, Notoginsenoside R1; PCAD, Protocatechuic aldehyde; RRP, Rg1-Notoginsenoside R1-Protocatechuic aldehyde; PFA, paraformaldehyde; L-FSS, Low-fluid shear stress; TC, total cholesterol; TG, triglyceride; HDL-C, high-density lipoprotein cholesterol; LDL-C, low-density lipoprotein cholesterol; AI, atherosclerosis index; ET-1, endothelin-1; PGI2, Prostaglandin I2: TXA2, Thromboxane A2; ECM, endothelial cell medium; RT, room temperature; SDS-PAGE, sodium dodecyl sulfate-polyacrylamide gel electrophoresis; PVDF, polyvinylidene fluoride.
